# Magnetic resonance imaging with upconversion nanoprobes capable of crossing the blood-cerebrospinal fluid barrier

**DOI:** 10.1186/s12951-024-02301-1

**Published:** 2024-01-30

**Authors:** Fang Han, Jiahao Gao, Guanglei Lv, Tao Liu, Qingfeng Hu, Meilin Zhu, Zunguo Du, Jing Yang, Zhenwei Yao, Xiangming Fang, Dalong Ni, Jiawen Zhang

**Affiliations:** 1 Department of Radiology, Huashan Hospital, Fudan University, Shanghai, 200040 P.R. China; 2 Department of Materials Science and State Key Laboratory of Molecular Engineering of Polymers, Fudan University, Shanghai, 200433 P.R. China; 3 Department of Oncology, Huashan Hospital, Fudan University, Shanghai, 200040 P.R. China; 4 Department of Urology, Huashan Hospital, Fudan University, Shanghai, 200040 P.R. China; 5 Department of Pathology, Huashan Hospital, Fudan University, Shanghai, 200040 P.R. China; 6 Department of Medical Imaging, The Affiliated Wuxi People’s Hospital of Nanjing Medical University, Wuxi, Jiangsu Province 214023 P.R. China; 7 Department of Orthopaedics, Shanghai Key Laboratory for Prevention and Treatment of Bone and Joint Diseases, Shanghai Institute of Traumatology and Orthopaedics, Ruijin Hospital, Shanghai Jiao Tong University School of Medicine, Shanghai, 200025 P.R. China

**Keywords:** Ventriculography, Upconversion nanoprobe, Blood-brain barrier, Blood-cerebrospinal fluid barrier, Magnetic resonance imaging

## Abstract

The central nervous system (CNS) maintains homeostasis with its surrounding environment by restricting the ingress of large hydrophilic molecules, immune cells, pathogens, and other external harmful substances to the brain. This function relies heavily on the blood-cerebrospinal fluid (B-CSF) and blood-brain barrier (BBB). Although considerable research has examined the structure and function of the BBB, the B-CSF barrier has received little attention. Therapies for disorders associated with the central nervous system have the potential to benefit from targeting the B-CSF barrier to enhance medication penetration into the brain. In this study, we synthesized a nanoprobe ANG-PEG-UCNP capable of crossing the B-CSF barrier with high targeting specificity using a hydrocephalus model for noninvasive magnetic resonance ventriculography to understand the mechanism by which the CSF barrier may be crossed and identify therapeutic targets of CNS diseases. This magnetic resonance nanoprobe ANG-PEG-UCNP holds promising potential as a safe and effective means for accurately defining the ventricular anatomy and correctly locating sites of CSF obstruction.

**Supplementary Information:**

The online version contains supplementary material available at 10.1186/s12951-024-02301-1.

## Introduction

Brain barrier structures, including blood cerebrospinal fluid (B-CSF) and the blood-brain barrier (BBB), are critical for maintaining the stability of the brain microenvironment and preventing hazardous substances from entering the brain [1]. The blood-brain barrier has been extensively studied in the diagnosis and treatment of central nervous system diseases. However, the B-CSF barrier has received little attention from fundamental neuroscientific researches. The B-CSF barrier is formed by choroid plexus (CP) epithelial cells, which are held together by tight junction proteins. The main function of the B-CSF barrier is to preserve cerebrospinal fluid (CSF) homeostasis, preventing harmful substances from entering the central nervous system (CNS) [2, 3]. The B-CSF barrier is essential for both the secretion of CSF and the exchange of hormones, nutrients, metal ions, and small molecules between the blood and the CSF [4]. Its biological function and potential value for central drug delivery have not been fully appreciated. Additionally, B-CSF barrier integrity is closely associated with CNS infections and other diseases. Therefore, it is important to develop noninvasively diagnostic method to maintain the integrity of the B-CSF barrier and CSF homeostasis.

In loculated hydrocephalus, especially multiloculated hydrocephalus (MLH) secondary to infection, CSF accumulates in isolated spaces due to the presence of septa. This poses a significant challenge for the diagnosis and treatment of infections. Particularly due to the difficulty in identifying an obstruction site, patients often require repeated shunt surgeries, which can cause severe psychomotor developmental disorders [5–7]. Understanding the anatomical structure and complex dynamics of the CSF is essential for effectively treating hydrocephalus, which relies heavily on ventriculography. However, traditional methods of ventriculography have some shortcomings that limit their clinical applicability, such as their high risk of large injuries and serious complications, long duration of treatment, and low degree of patient compliance. Therefore, developing a novel non-invasive imaging method for the B-CSF barrier is of great significance.

Molecular imaging provides significant advantages for targeted therapy and the early diagnosis and detection of various brain diseases, especially malignant cerebral tumors [8, 9]. However, few studies have examined the application of multifunctional nanoprobes in ventriculography. A major obstacle for applying molecular imaging to improve ventriculography is our limited knowledge of enzymes and transporters expressed on the B-CSF barrier [4, 10]. Receptor-mediated transcytosis (RMT) is an efficient pathway that has been shown to facilitate the translocation of nanoprobes across the BBB barrier [11–14]. Due to their potential usefulness as probes for molecular imaging, paramagnetic gadolinium (Gd^3+^)-ion-doped upconversion nanoparticles (UCNPs) have garnered increasing attention. In particular, use of nanoparticles allows for simultaneous phase and size control after doping in a UCNP matrix [15]. We previously synthesized a UCNP nanoprobe that crossed the BBB due to the attachment of Angiopep-2 (ANG, TFFYGGSRGKRNNFKTEEY), which was capable of binding specifically to the LRP receptor [16]. In addition to being highly expressed in the BBB, LRP is expressed in the B-CSF barrier [4]. Therefore, we speculated that these nanoparticles could cross the B-CSF barrier to facilitate non-invasive ventriculography.

In this study, an ANG label was added to the UCNP surface using a polyethylene glycol (PEG) linker [12] to create ANG-PEG-UCNP capable of successfully crossing the B-CSF barrier. ANG was used as a targeting ligand for the nanoprobe due to its effectiveness in facilitating transcytosis across the BBB and specific affinity for the LRP receptor [17–21]. Features of the ANG-PEG-UCNP allow it to traverse both the BBB and B-CSF barrier for noninvasive ventriculography. This task presents considerable challenges, may potentially improve the treatment of CNS infections, hydrocephalus, CSF rhinorrhea, and other neurological diseases. More importantly, ANG-PEG-UCNP may be used to effectively identify the anatomical structure of the ventricles and site of CSF obstruction by MRI, benefiting the diagnosis and treatment of CNS diseases and improving the quality of life of patients.

## Materials and methods

### Materials

MeO-PEG5k-SH was purchased from Jenkem Co., Ltd. Ammonium fluoride (NH_4_F), YbCl_3_·6H_2_O, YCl_3_·6H_2_O, TmCl_3_, 1-Octacene (90%), GdCl_3_·6H_2_O and N-hydroxysuccinimide (NHS) were purchased from Sigma Aldrich Reagent Co., Ltd. ANG (TFFYGGSRGKRNNFKTEEY) was obtained from Huashan Hospital, Fudan University. Reagents and chemicals used required no additional purification because they were of analytical grade.

### Cytotoxicity assessment

The cytotoxicity of the synthesized ANG-PEG-UCNP against Z310 cells was examined in vitro. In this study, Z310 cells were initially co-cultivated with 1000–15.62 µg/m concentrations of ANG-PEG-UCNP. Co-cultivation was carried out dose-dependently under controlled conditions (37 °C and 5% CO_2_). As described in the Materials section, 1% penicillin/streptomycin and 10% fetal bovine serum (FBS) were added to the DMEM culture medium. Subsequently, cells at an initial count of 104 cells/well were seeded in 96-well plates and placed in a controlled incubation chamber at 37 °C with 5% CO_2_ for 24 h. Following incubation, absorbance at a wavelength of 490 nm was measured using a Bio-TekelX 800 microplate reader to assess cellular and metabolic activity.

Three groups of five healthy Institute of Cancer Research (ICR) mice (each weighing approximately 35 g) were used. The experimental group of ICR mice were intravenously injected with 100 µL of ANG-PEG-UCNP, while the control group of mice received 100 µL of PBS. In vivo toxicities of injected materials were evaluated after 3 and 30 days. Histological analysis of dissected mouse liver (with and without staining with hematoxylin and eosin [H&E]), cardiac muscle, spleen, and lung tissues was performed. Blood samples were extracted from mice to evaluate toxicity.

### Assessment of B-CSF barrier permeability to ANG-PEG-UCNP

Human glioblastoma-derived U87MG and rat choroid plexus-derived Z310 cell lines were cultured in Dulbecco’s Modified Eagle Medium (DMEM) medium supplemented with 10% fetal bovine serum, 100 U/mL penicillin, and 100 µg/mL streptomycin. Once every 3 days, when cell density reached 80%, the medium was changed in a Thermo cell culture incubator set to 37ºC and 5% carbon dioxide. After digestion with 0.25% trypsin-ethylenediaminetetraacetic acid, cells were centrifuged to facilitate passage. To assess the specific ability of ANG to cross the B-CSF barrier, viable Z310 cells were co-cultured with ANG-PEG-UCNP, PEG-UCNP, Gd-DTPA, and iohexol (iodohexol) for 1 h. To observe the phagocytic response of cells to different contrast agents, cells were treated with 4’,6-diamidino-2-phenylindole (DAPI) and photographed using confocal laser scanning microscopy. In addition, co-incubation times of two types of nanoprobes, ANG-PEG-UCNP and PEG-UCNP, with the Z310 cell line were assessed to evaluate the ability of probes to cross the B-CSF barrier and assess corresponding effects.

### In vitro B-CSF barrier model

Z310 cells were used to create an in vitro model of the B-CSF barrier. Cells were cultured on 24-well polycarbonate transwell membranes with an average pore diameter of 1.0 μm and a surface area of 0.33 cm^2^. A cell density of 5 × 10^4^ cells per well was ensured using a FALCON Cell Culture Insert (Becton Dickinson Labware, USA). An epithelial voltmeter (Millicell-RES, Millipore, USA) was used to evaluate the integrity of cell monolayers. Tests included trans-endothelial electrical resistance (TEER) values exceeding 200 Ω cm^2^. Further, ANG-PEG-UCNP (Gd 10 µg/mL) were introduced to the apical side chamber of the B-CSF barrier model, which was equivalent to the blood side in vivo. The mixture was shaken gently at 50 rpm. Following an 18-hour incubation period, the cell filter membrane was separated from the support, and basolateral media were removed to measure Gd content via ICP-OES. Identical protocols were used to evaluate PEG-UCNP controls of the same concentrations. In addition, a blocking experiment was conducted in which free ANG (3 mg/mL) was added. Complexes were removed from the transwell following a 30-minute incubation period. Subsequently, 300 µg/mL ANG-PEG-UCNP (Gd 10 µg/mL) along with 3 mg/mL ANG were introduced before following above procedures.

### Probe phagocytosis evaluation via a cellular fluorescence assay

In a specially designed laser confocal cell culture dish, the inoculum density of the Z310 cell line was adjusted. Thereafter, cells were incubated for 24 h. After adjusting to a concentration of 750 µg/mL, ANG-PEG-UCNP and PEG-UCNP were distributed in RPMI 1640/DMEM medium and added to culture dishes. In addition, Gd-DTPA with an iodohexol contrast agent modified with fluorescein isosulfate (FITC) were dissolved in DMEM to compare levels of phagocytosis of new nanoprobes and currently used clinical imaging probes capable of crossing the B-CSF barrier in vitro. Cells were rinsed three times with PBS to remove any nanoparticles not taken up by cells following a 1-hour co-incubation with the above-mentioned probes. Cellular nuclei were stained with DAPI. Confocal fluorescence imaging tests were performed in which the excitation wavelength for DAPI was 358 nm. The luminescence signal was identified within a 400–500 nm wavelength range and visualized using the 60× oil immersion objective. Immediately thereafter, we evaluated the effect of nanoprobe-cell co-incubation time on probe phagocytosis. ANG-PEG-UCNP and PEG-UCNP were co-incubated with the cells for 30, 60, and 120 min. In addition, we evaluated the effect of probe concentration on nanoprobe uptake by Z310 cells. Coincubations (for 60 min at 37 °C) of Z310 cell lines with different concentrations of ANG-PEG-UCNP and PEG-UCNP (1500, 750, 375, 200 µg/mL) were performed. We also evaluated the impact of incubation temperature on the uptake of ANG-PEG-UCNP in the Z310 cell line species by co-incubating cells and probes at two temperatures (5 and 37 °C).

The mechanism by which barrier closure occurs was evaluated by pre-incubating the culture plates with an ANG blocker (3 mg/mL). After co-incubation of the blocker with Z310 cells at 37 °C and 5% CO_2_ for 0.5 h, 750 µg/mL ANG-PEG-UCNP were added. To complete experiments, steps described previously were repeated.

### In vivo MR imaging and ventriculography

Male Balb/c nude mice used in this study were obtained from the Chinese Academy of Sciences Laboratory Animal Center. At 6–8 weeks of age, mice weighed an average of 20 g. The right striatum (0.6 mm anterior to the bregma, 1.8 mm laterally, and 3.0 mm in depth) was injected with U87MG cells (5.0 × 10^5^ cells immersed in 5 µL of phosphate-buffered saline (PBS) using a stereotactic fixation apparatus equipped with a specialized adaptor designed for mice. In vivo MR experiments with ANG-PEG-UCNP were performed when the tumors reached approximately 0.5–1 mm in length (3–4 weeks after inoculation), causing the midline structure to shift and forming the hydrocephalus model. Animal studies were conducted in accordance with guidelines of the Institutional Animal Care and Use Committee.

In vivo MRI tests were performed using a Siemens 3.0 T Tim Trio MRI apparatus. Mice were anesthetized with chloral hydra and placed on a mouse bed. Mice brain *T*_*1*_-weighted images were obtained prior to contrast administration and 0, 0.5, 1, 2, 6, 12, 24, and 48 h after intravenous tail-vein administration of Gd-DTPA, PEG-UCNP, or ANG-PEG-UCNP (6 mg Gd/Kg dose, an equivalent amount, 150 µL per mouse). MR coronal images were performed vertically to the anterior-posterior (long) axis of the animal using a *T*_*1*_-weighted sequence under the following specified conditions: repetition time, 400 ms; echo time, min full; minimum TE, 14.2 ms; maximum TE, 28.4 ms; echo train length, 2; flip angle, 142°; NEX, 8; field of view (fov), 60 mm; fov phase, 1; slice thickness, 1 mm; number of slices, 10; and bandwidth, 25 Hz/Px. To investigate the transportation of nanoparticles across the B-CSF barrier and assess the specific impact of ANG-PEG-UCNP on Z310 cells in vivo, cells of the same lateral ventricle were obtained before and after the administration of ANG-PEG-UCNP, PEG-UCNP, or Gd-DTPA. Software from Siemens Medical Systems was used to measure signal intensities using regions of interest inside the ventricle of the same size.

### Detection of gadolinium content in brain tissue

We investigated the ability of ANG-mediated UCNP to cross the B-CSF barrier in the mouse hydrocephalus model implanted with glioblastoma cells. To further confirm the ability of the ANG-PEG-UCNP to traverse the B-CSF barrier during MRI, we intravenously infected mice with 6 mg Gd/kg (150 µL of, *n* = 3). Mice were sacrificed after receiving injections of Gd-DTPA, ANG-PEG-UCNP, or PEG-UCNP for one hour each. Brain tissue was ground near the cerebrospinal fluid and dissolved in hydrochloric acid. The Gd content of local brain tissue was determined using inductively coupled plasma (ICP-OS). Gd retention values within local brain tissue after injection with the three different contrast agents were compared.

### Statistical analysis

All data analyses in this article were performed using graphpad prism 8.0 software. Mean ± SD values are used to describe numerical data. To examine between-group differences, a two-tailed adjusted Student’s t-test was used. Values of *p* < 0.05 were considered significant.

## Results and discussion

### Synthesis and characterization of ANG-PEG-UCNP

**Scheme 1:**
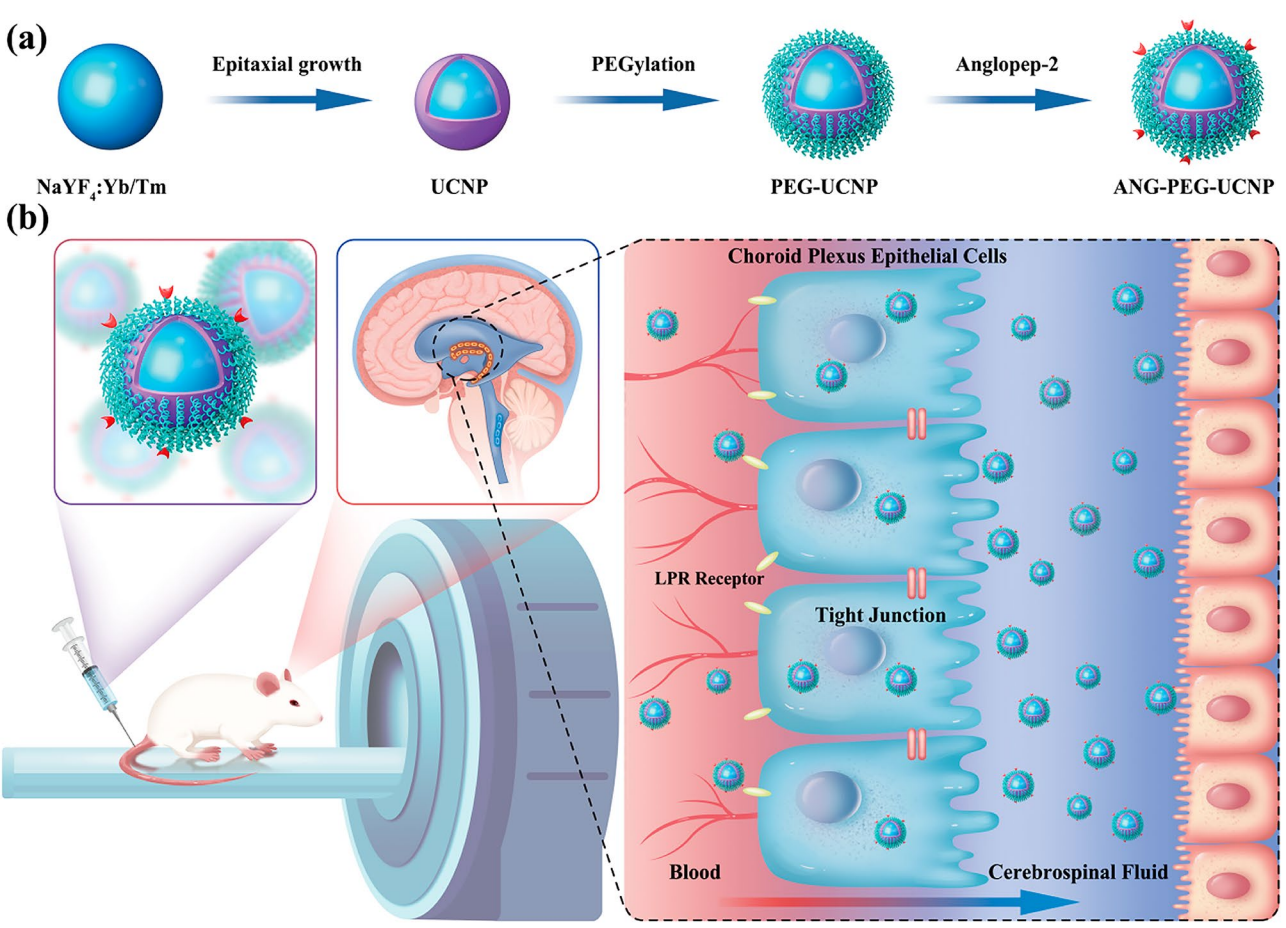
Design of ANG-PEG-UCNP for ventriculography. **(a)** ANG-PEG-UCNP synthesis. (**b)** Schematic illustration of ANG-PEG-UCNP binding to LRP receptors for B-CSF barrier crossing *via* LRP receptor-mediated endocytosis

**Fig. 1:**
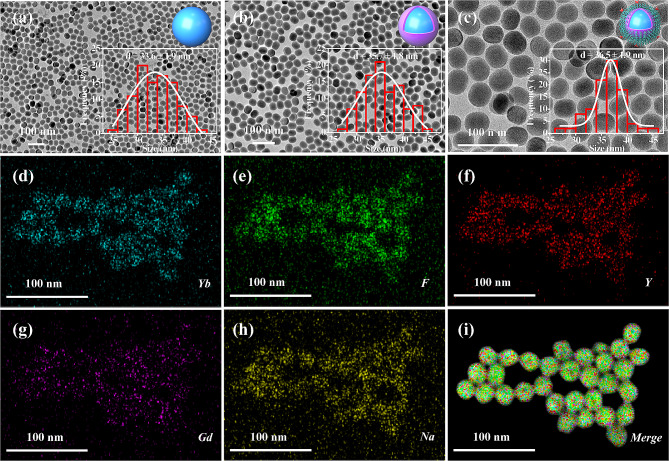
Transmission electron microscopy (TEM) images of **(a)** core upconversion nanoparticles (UCNP, NaYF4:Yb/Tm), **(b)** core-shell UCNP (NaYF_4_:Yb/Tm@NaGdF_4_), and **(c)** Angiopep-2/polyethylene glycol-UCNP (ANG-PEG-UCNP). **(d-i)** Element mappings of basic chemical elements (Yb, F, Y, Gd, and Na) of ANG-PEG-UCNP.

The preparation of ANG-PEG-UCNP is illustrated in Scheme 1a. First, UCNPs (NaYF_4_: Yb/Tm@NaGdF_4_) are coated with oleic acid (OA) ligands and synthesized using a method previously reported [16]. Obtained UCNPs were highly monodisperse and morphologically homogeneous. The incorporation of a coated ultrathin NaGdF_4_ shell significantly improved both the upconversion luminescence of UCNPs and MRI performance [15, 16]. Next, OA was removed using hydrochloric acid to incorporate hydrophobic UCNP within the aqueous phase [22]. OA-free UCNP were then coated with amine-poly (ethylene glycol)-thiol (NH_2_-PEG_5k_-SH) to obtain PEG-UCNP via intense thiol-metal binding [23]. Ultimately, necessary PEG-UCNP was produced via covalent interactions between amino groups of PEG-UCNP and the carboxyl groups of ANG [18]. Transmission electron microscopy (TEM) images (Fig. 1a,b) revealed that NaYF_4_:Yb/Tm and NaYF_4_:Yb/Tm@NaGdF_4_ displayed uniform spherical nanoparticles with well-defined dimensions. The average particle size of hydrophilic ANG-PEG-UCNP was 36.5 nm with the main particle-size-distribution interval (36 ~ 38 nm, 30%) (Fig. 1c). With the gradual coating of ultrathin NaGdF_4_ shell and ANG-PEG layers on the UCNP, the average particle size of ANG-PEG-UCNP nanocomposite gradually increases. The NaGdF_4_ shell had a thickness of approximately 1 nm, which is considered optimal for well-balanced MR performance [12]. Element mappings confirmed the existences of various elements (F, Y, Na, Gd, and Yb) (Fig. 1d–i).

**Fig. 2:**
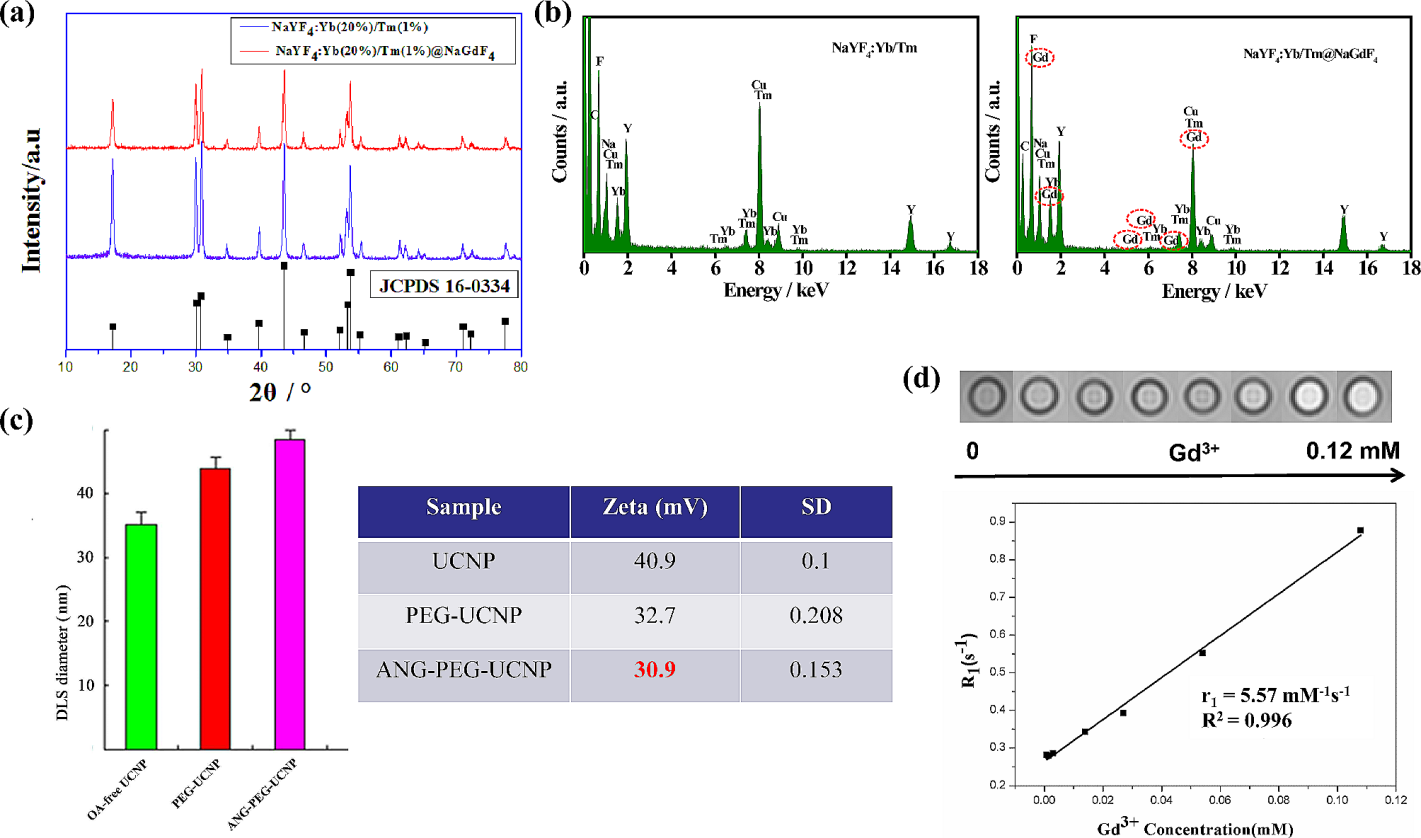
**(a)** X-ray diffraction (XRD) patterns of NaYF_4_:Yb/Tm (top) and NaYF_4_:Yb/Tm@NaGdF_4_ (bottom). **(b)** Energy-dispersive X-ray (EDX) spectra of NaYF_4_:Yb/Tm and NaYF_4_:Yb/Tm@NaGdF4. **(c)** Dynamic light scattering (DLS) sizes (intensity-based) (left) and zeta potentials (right) of oleic acid-free UCNP, PEG-UCNP, and ANG-PEG-UCNP, respectively. **(d)***T*_*1*_-weighted magnetic resonance images of ANG-PEG-UCNP with different Gd^3+^ concentrations and R1 plots against Gd3 + concentrations

X-ray powder diffraction (XRD) spectra revealed the excellent crystallinity and hexagonal phase structure ofANG-PEG-UCNP (Fig. 2a). The fundamental components of the ANG-PEG-UCNP were further revealed by energy-dispersive X-ray (EDX) spectroscopy (Na, Y, F, Yb, Tm, and Gd) (Fig. 2b). OA-UCNPs were then transferred from cyclohexane to water using a previously reported method, with minor changes [24]. Dynamic light scattering (DLS) revealed that nanoprobes were uniformly dispersed in water without aggregation. Hydrodynamic diameter (HD) values determined via DLS (Fig. 2c, Supplemental Fig. S1) increased due to the large hydrodynamic volume of PEG [24]. Additionally, HD values of ANG-PEG-UCNP slightly increased. This increase was most likely caused by the attachment of ANG to the surface of the PEG-UCNP. All the above cases had low polydispersity indices (PDI), indicating that the NPs were perfectly dispersed in water. Zeta potential measurements of the preparation further suggested the successful conjugations of the PEG chains and ANG molecules (Fig. 2c).

The synthesized ANG-PEG-UCNP was subjected to *T*_*1*_-weighted MRI using a 3.0-T MRI scanner. Results demonstrated a concentration-dependent reinforcement effect, a finding supported by a specific longitudinal relaxivity (r_1_) value of 5.57 mM^− 1^s^− 1^(Fig. 2d). This enhancement was primarily due to the suppression of a “negative lattice shield effect”(n-LSE) by the ultrathin NaGdF_4_ shell layer [12]. Moreover, in contrast to the rapid renal clearance observed for Gd-DTPA, ANG-PEG-UCNP circulation lifetime was increased through PEGylation. We also provided the *T*_*2*_ relaxation properties of ANG-PEG-UCNP (Supplemental Fig. S2).

### Cytotoxicity and cellular uptake studies

**Fig. 3:**
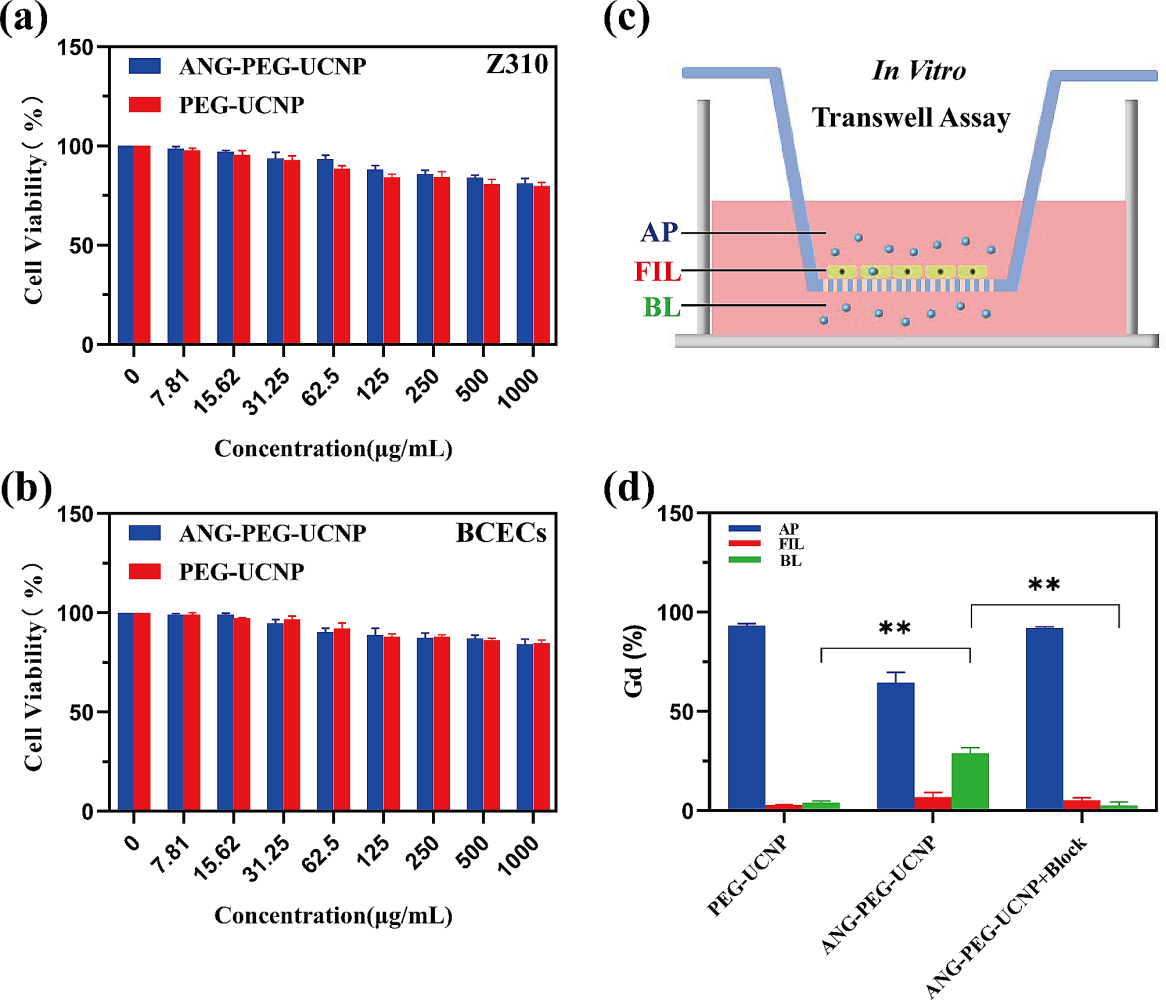
In vitro viability of **(a)** Z310 cells and **(b)** BCECs cultured with PEG-UCNP or ANG-PEG-UCNP for 24 h. **(c)** An illustration of the Z310 transwell assay used to assess whether nanoprobes crossed the blood cerebrospinal fluid barrier. **(d)** Corresponding transcytosis of PEG-UCNP and ANG-PEG-UCNP without or with ANG blockage. (**p < 0.01)

The cytotoxicity of ANG-PEG-UCNP was investigated in Z310 cells in vitro using a classic 3-(4,5-dimethylthiazol-2-yl)-2,5-diphenyl-2 H-tetrazolium bromide (MTT) assay. Cell viability remained above 80% 24 h after treatment with ANG-PEG-UCNP at concentrations ranging from 0 to 1000 µg/mL (Fig. 3a). MTT analyses showed that < 24 h of treatment with higher concentration of ANG-PEG-UCNP (1,000 µg/mL) produced no obvious cell cytotoxicity, demonstrating a lack of toxicity of ANG-PEG-UCNP in cells in vitro. In addition, the probes were also not significantly cytotoxic in brain capillary endothelial cells (BCECs) (Fig. 3b).

To examine the in vivo toxicity of ANG-PEG-UCNP, ICR mice were intravenously injected with ANG-PEG-UCNP (experimental group) or PBS (control group). No weight abnormalities were observed in mice throughout a 30-day observation period (Supplemental Fig. S3). Further, no significant variations in biochemical parameters of blood were observed. H&E staining showed that ANG-PEG-UCNP had good biocompatibility with the heart, spleen, liver, lungs, kidneys, and other organs (Supplemental Fig. S4).

Further, we constructed an in vitro BCSFB model by Transwell chamber and evaluated the transmembrane permeability of ANG-PEG-UCNP (Fig. 3c). PEG-UCNP or ANG-PEG-UCNP was added to the cells in the upper compartment for incubation. The Gd content in the lower compartment was detected using the ICP-OES method after 18 h. Compared to that of cells treated with PEG-UCNP, uptake of ANG-PEG-UCNP by Z310 cells was increased (Fig. 3d), revealing that the presence of the ANG peptide on the PEG-UCNP surface may facilitate the transport of ANG-PEG-UCNP into cells. This also indicated that the ANG ligand interacts with the LRP receptor, as further confirmed *via* a blocking study. To assess the specificity of ANG receptor targeting, Z310 cells were subjected to ANG-PEG-UCNP treatment while high-dose ANG was concurrently used as a blocking agent. After treatment with a blocking agent, Z310 cells showed minimal cellular uptake of ANG-PEG-UCNP (Fig. 3d). These results provide initial evidence showing that the ANG peptide facilitates the cellular uptake of UCNP. Furthermore, cellular uptake of ANG-PEG-UCNP by Z310 cells was significantly improved by specifically targeting the LRP receptor via the ANG peptide.

### Cellular mechanism of ANG-PEG-UCNP for crossing the B-CSF barrier

**Fig. 4:**
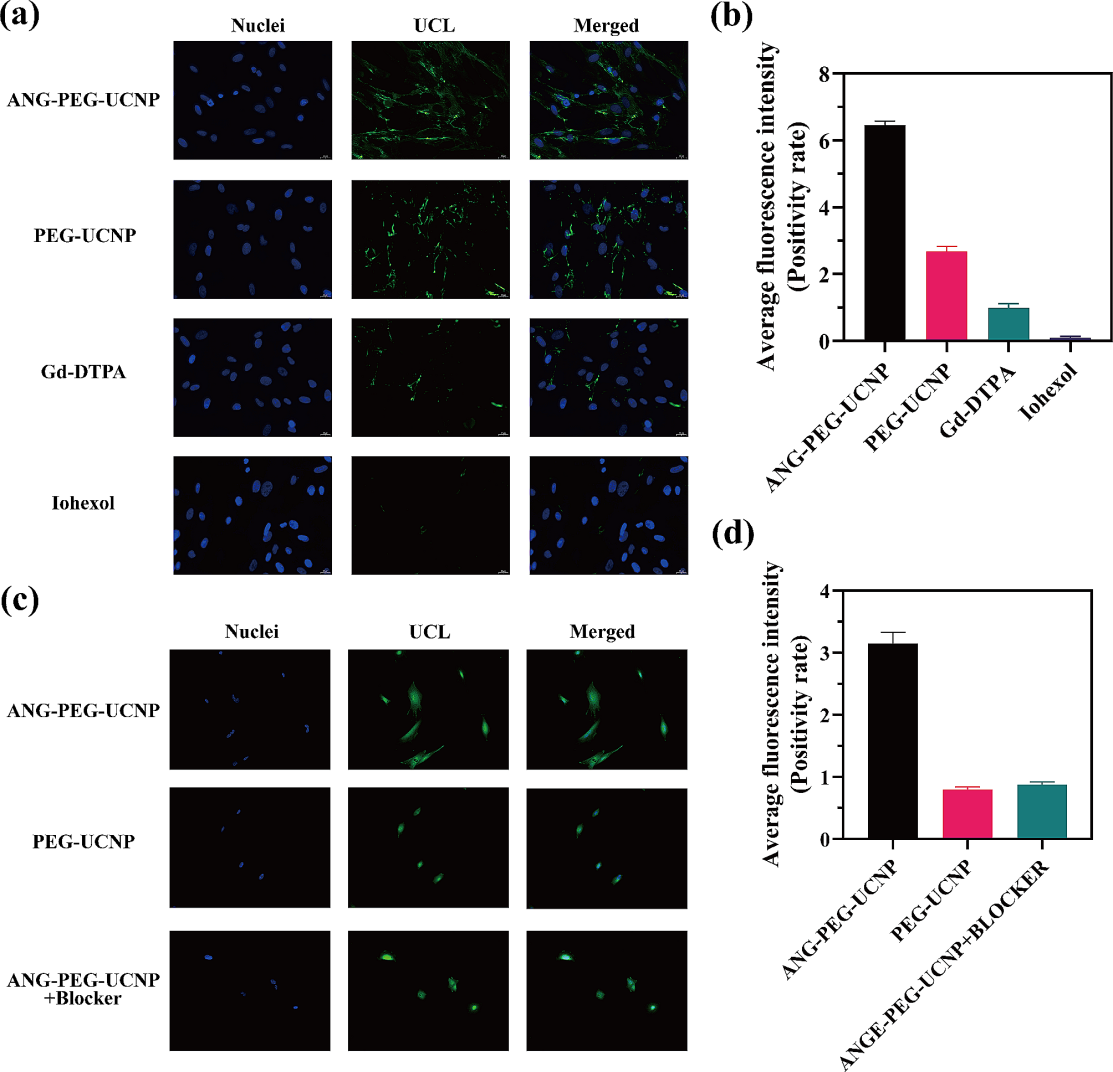
**(a)** Typical confocal UCL images of Z310 cells cultured with ANG-PEG-UCNP, PEG-UCNP, Gd-DTPA with fluorescein isosulfate (FITC), and iohexol with FITC. **(b)** Fluorescence distributions of ANG-PEG-UCNP, PEG-UCNP, Gd-DTPA with FITC, and iohexol with FITC. **(c)** Typical confocal UCL images of Z310 cells treated with ANG-PEG-UCNP, PEG-UCNP, and ANG-PEG-UCNP after blocking with free ANG. All samples were standardized at a concentration of 300 µg/mL. (**d)** Distribution of fluorescence after treatment with ANG-PEG-UCNP, PEG-UCNP, and ANG-PEG-UCNP after blocking with free ANG.

At the cellular level, we assessed receptor targeting by nanoprobes and the degree to which new probes crossed the barrier between the CSF and blood. As shown in Fig. 4a, levels of phagocytosis of different probes by choroid plexus epithelial cells were examined by co-culturing rat choroid plexus Z310 cells with nanoprobes or currently used clinical MRI and computed tomography contrast agents. The results showed that the ANG-PEG-UCNP was taken up by Z310 cells at higher levels than PEG-UCNP, Gd-DTPA, and iodine contrast agents. Quantitative analysis (Fig. 4b) revealed that PEG-UCNP and Gd contrast agents were also partially taken up by Z310 cells. This uptake may be related to leakage from the cell surface and the secretion of surface receptors. Clinical contrast agents including Gd-DTPA and ioxehl labeled with FITC were less taken up by the Z310 cell line, suggesting that conventional clinical contrast agents are not useful for visualizing the B-CSF barrier. Furthermore, effects of cell-nanoprobe coincubation time on the efficiency by which ANG-PEG-UCNP and PEG-UCNP crossed the B-CSF were evaluated in Z310 cells. Results indicated that ANG-PEG-UCNP uptake was maximal after 60 min of co-incubation with Z310 cells (Supplemental Fig. S5a). In contrast, the PEG-UCNP did not exhibit similar functional properties, suggesting that the absence of ANG targeting to the B-CSF barrier may account for this discrepancy (Supplemental Fig. S5b). In addition, levels of ANG-PEG-UCNP uptake were higher than those of PEG-UCNP in Z310 cells, a phenomenon that may be due to targeted interactions between ANG and LRP.

We also found that ANG-PEG-UCNP and PEG-UCNP concentrations affected cellular uptake. Specifically, higher concentrations of nanoprobes exhibited enhanced cellular uptake (Fig. S5c, S5d). Furthermore, incubation temperature influenced the uptake of ANG-PEG-UCNP by Z310 cells, with cellular uptake levels increased at 37 ℃ (Supplemental Fig. S6a-b). In addition, when Z310 cells were pretreated with an ANG-specific blocker and subsequently incubated with ANG-PEG-UCNP, a significantly diminished fluorescence signal was observed (Fig. 4c). This finding provides additional evidence supporting the hypothesis that the enhanced uptake of ANG-PEG-UCNP in Z310 cells is mainly mediated by their specific binding to the LRP receptor (Fig. 4d).

### Blood-cerebrospinal fluid barrier permeability in vivo and the detection of gadolinium in brain tissue

**Fig. 5:**
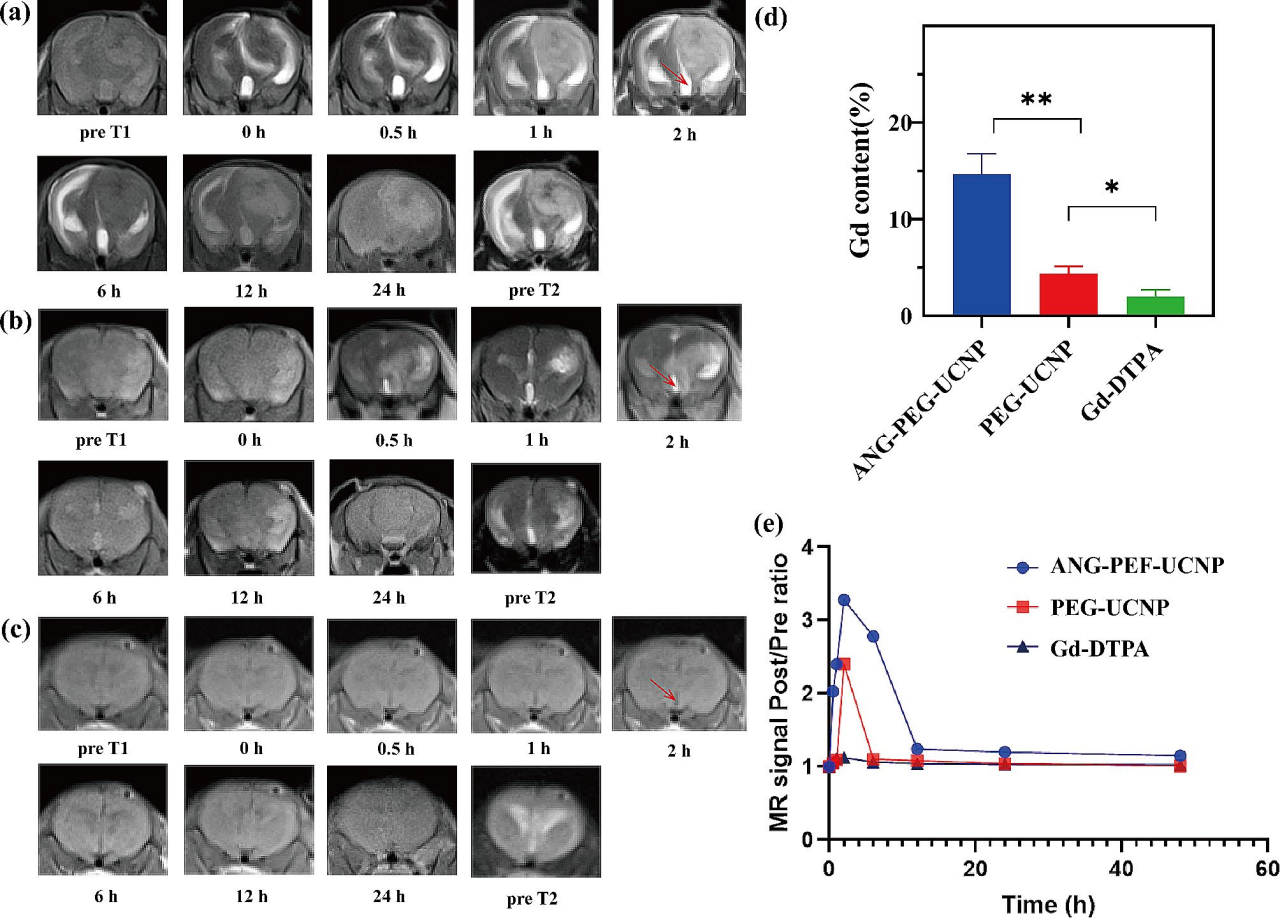
**(a–c)***T*_2_- and *T*_1_-weighted MR images of mice after intravenous injection of ANG-PEG-UCNP, PEG-UCNP, and Gd-DTPA at selected time points. **(d)** Brain tissue Gd content of different groups measured by ICP-OES at 2 h after ANG-PEG-UCNP, PEG-UCNP, and Gd-DTPA injection, respectively. **(e)** Time-dependent post/pre-ratio values of different groups. (*p < 0.05,**p < 0.01)

H&E-stained brain tissues from mice confirmed the successful establishment of hydrocephalus model caused by glioma (Supplemental Fig. S7). *T*_*1*_- and *T*_*2*_-weighted MRI scans were performed before injecting nanoprobes to confirm that the hydrocephalus model had been successfully created. Dynamic *T*_*1*_-weighted MRI was performed 0, 0.5, 1, 2, 6, 12, and 24 h post injection of ANG-PEG-UCNP, PEG-UCNP, or clinical Gd-DTPA via the tail vein. As shown in Fig. 5a–c, obvious cerebral ventricle dilation was observed on *T*_*2*_-weighted imaging (T_2_WI), confirming the successful establishment of the hydrocephalus model. The *T*_*1*_-weighted MR signal intensity of the ventricular system was dramatically increased in cells treated with ANG-PEG-UCNP versus PEG-UCNP and Gd-DTPA. It is worth noting that after ANG-PEG-UCNP administration, the ratio of ventricular system signal intensity post-injection versus pre-injection (Post/Pre ratio) instantly rose, reaching a maximum value of 3.28 at 2 h post-injection. *T*_*1*_-weighted MR revealed that ANG-PEG-UCNP had a longer circulatory lifespan (> 6 h) than other molecules assessed. In comparison, the maximal post-/pre-ratio obtained after PEG-UCNP administration was 2.4 at 2 h post-injection. Due to the presence of ample vasculature around the tumor margin, the peritumoral distribution of PEG-UCNP can be explained by nanoparticle extraversion due to increased enhanced permeability and retention (EPR) effect. Minimal reinforcement was observed in the Gd-DTPA group. To ensure the reliability of these results, we performed ICP-OES on brain tissues taken from hydrocephalus model mice 2 h after injection with different probes. The results showed that the brain tissues of the animals injected with ANG-PEG-UCNP had significantly higher Gd content than the other two groups. This suggests a significant uptake of ANG-PEG-UCNP by B-CSF barrier, which is also consistent with our results obtained in *T*_*1*_-MRI image analysis (Fig. 5d).

As shown in Fig. 5e, Gd content of local brain tissue was increased in mice after injection with ANG-PEG-UCNP, suggesting the excellent brain barrier-targeting ability of ANG. This finding provided a scientific basis for facilitating imaging by targeting the B-CSF barrier with ANG-linked nanoprobes. We further plotted the histograms of MR signal post/pre ratios after 2 h of different probe injections, and the results showed that the MRI parameters of post/pre in the ventricular region of the ANG-PEG-UCNP - injected group were significantly higher than those of the PEG-UCNP group and the Gd-DTPA group (Supplemental Fig. S8). Use of a PEG-UCNP nanoprobe lacking the ANG-targeting ligand resulted in reduced Gd retention in local brain regions; however, the PEG-UNCP resulted in Gd retention levels that were higher than those associated with the use of a clinical Gd-DTPA probe. This may be related to the excellent size characteristics of the nanoprobe and brain barrier leakage due to the glioma. The local retention rate of clinical Gd-DTPA nanoprobes is low, which may be due to their extracellular gap distribution properties and high rates of metabolism.

## Conclusion

In this study, a dual-targeting nanoprobe (i.e., ANG-PEG-UCNP) was developed to facilitate transport across the B-CSF barrier via RMT. ANG-PEG-UCNP improved the feasibility of non-invasive ventriculography via MRI and enhanced the visualization of ventricular system anatomy due to the targeting specificity and high sensitivity of the probes. Use of ANG-PEG-UCNP may potentially provide a non-invasive method for understanding unique anatomical features and complex cerebrospinal fluid dynamics in patients with hydrocephalus. Overall, it is believed that the MRI nanoprobe may increase the safety and effectiveness of diagnostic and therapeutic methods in patients with hydrocephalus. More importantly, risk of infection and dysfunction is reduced due to conventional repeated shunt surgeries may be reduced due to the creation of ANG-PEG-UCNP.

### Electronic supplementary material

Below is the link to the electronic supplementary material.

Supplementary Material 1

## Data Availability

No datasets were generated or analysed during the current study.
