# Supplementary material for: Magnetic resonance imaging with upconversion nanoprobes capable of crossing the blood-cerebrospinal fluid barrier
Source: J Nanobiotechnology. 2024 Jan 30;22:43. doi: 10.1186/s12951-024-02301-1 (PMC10826186; doi:10.1186/s12951-024-02301-1)
Supplement: Supplementary file 1 — Supplementary Material 1 [file 12951_2024_2301_MOESM1_ESM.doc]

**Additional file 1**

**Magnetic Resonance Imaging with Upconversion Nanoprobes Capable of Crossing the** **Blood-Cerebrospinal Fluid Barrier**

Fang Han^1†^, Jiahao Gao^1†^, Guanglei Lv^2†^, Tao Liu^3^, Qingfeng Hu^4^, Meilin Zhu^1^, Zunguo Du^5^, Jing Yang^1^, Zhenwei Yao^1^, Xiangming Fang^6^*, Dalong Ni^7^*, Jiawen Zhang^1^*

^1^ Department of Radiology, Huashan Hospital, Fudan University, Shanghai 200040, P.R. China.

^2^Department of Materials Science and State Key Laboratory of Molecular Engineering of Polymers, Fudan University, Shanghai 200433, P.R. China.

^3^ Department of Oncology, Huashan Hospital, Fudan University, Shanghai 200040, P.R. China.

^4^Department of Urology, Huashan Hospital, Fudan University, Shanghai 200040, P.R. China.

^5^Department of Pathology, Huashan Hospital, Fudan University, Shanghai 200040, P.R. China.

^6^Department of Medical Imaging, The Affiliated Wuxi People’s Hospital of Nanjing Medical University, Wuxi 214023, Jiangsu Province, P.R. China.

^7^Department of Orthopaedics, Shanghai Key Laboratory for Prevention and Treatment of Bone and Joint Diseases, Shanghai Institute of Traumatology and Orthopaedics, Ruijin Hospital, Shanghai Jiao Tong University School of Medicine, Shanghai 200025, P.R. China.

Fang Han, Jiahao Gao, and Guanglei Lv contributed equally to this work.

* Corresponding author

Prof. Jiawen Zhang

Email: jiawen_zhang@fudan.edu.cn

Prof. Dalong Ni

Email: ndl12353@rjh.com.cn

Prof. Xiangming Fang

Email: xiangming_fang@njmu.edu.cn

**Fig. S1** DLS sizes of PEG-UCNP and ANG-PEG-UCNP (intensity-based).


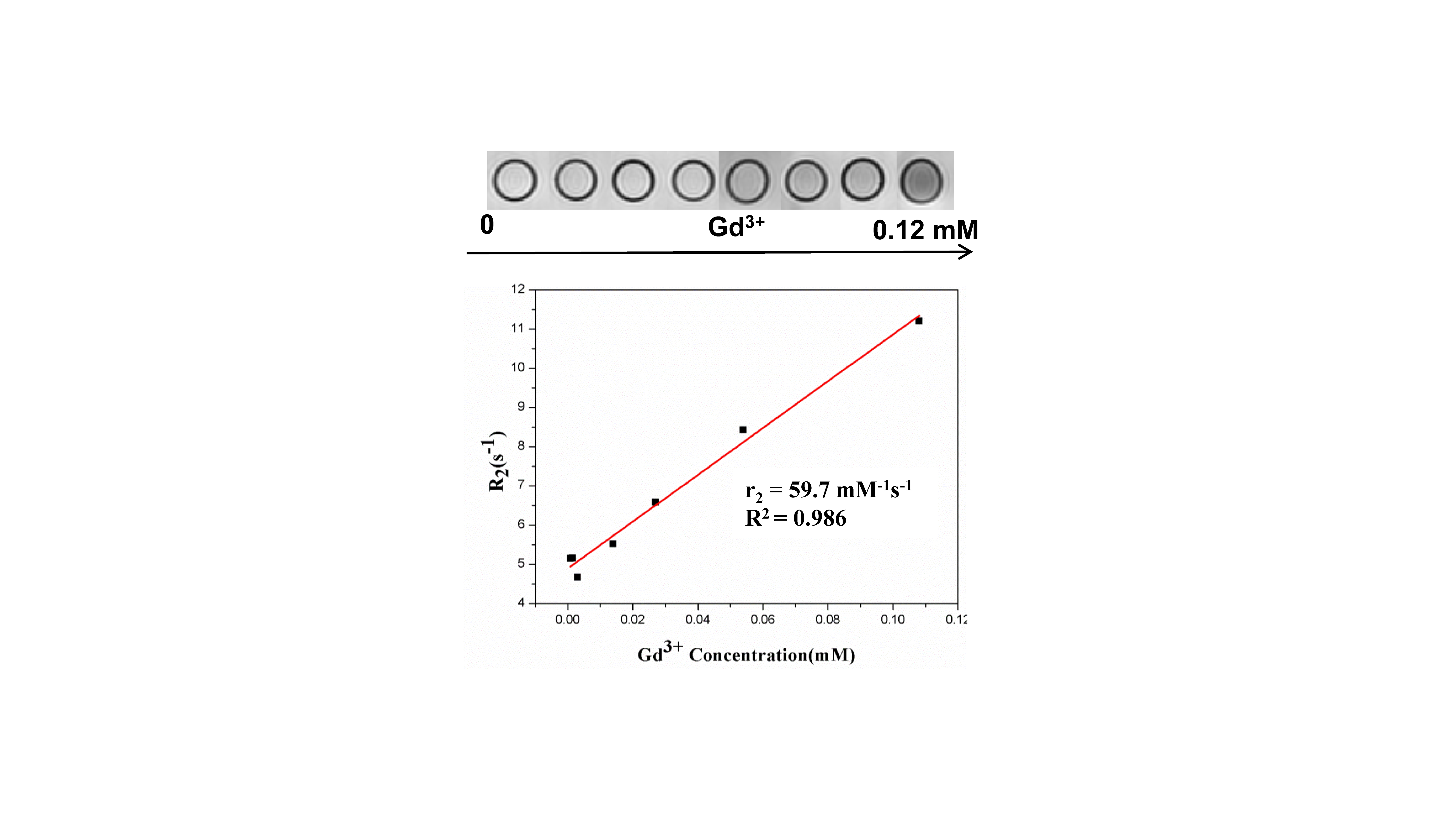


**Fig. S2** T_2_-weighted MR images of ANG-PEG-UCNP with different Gd^3+^ concentrations (upper) and plots of R_2_ versus Gd^3+^ concentrations (bottom).


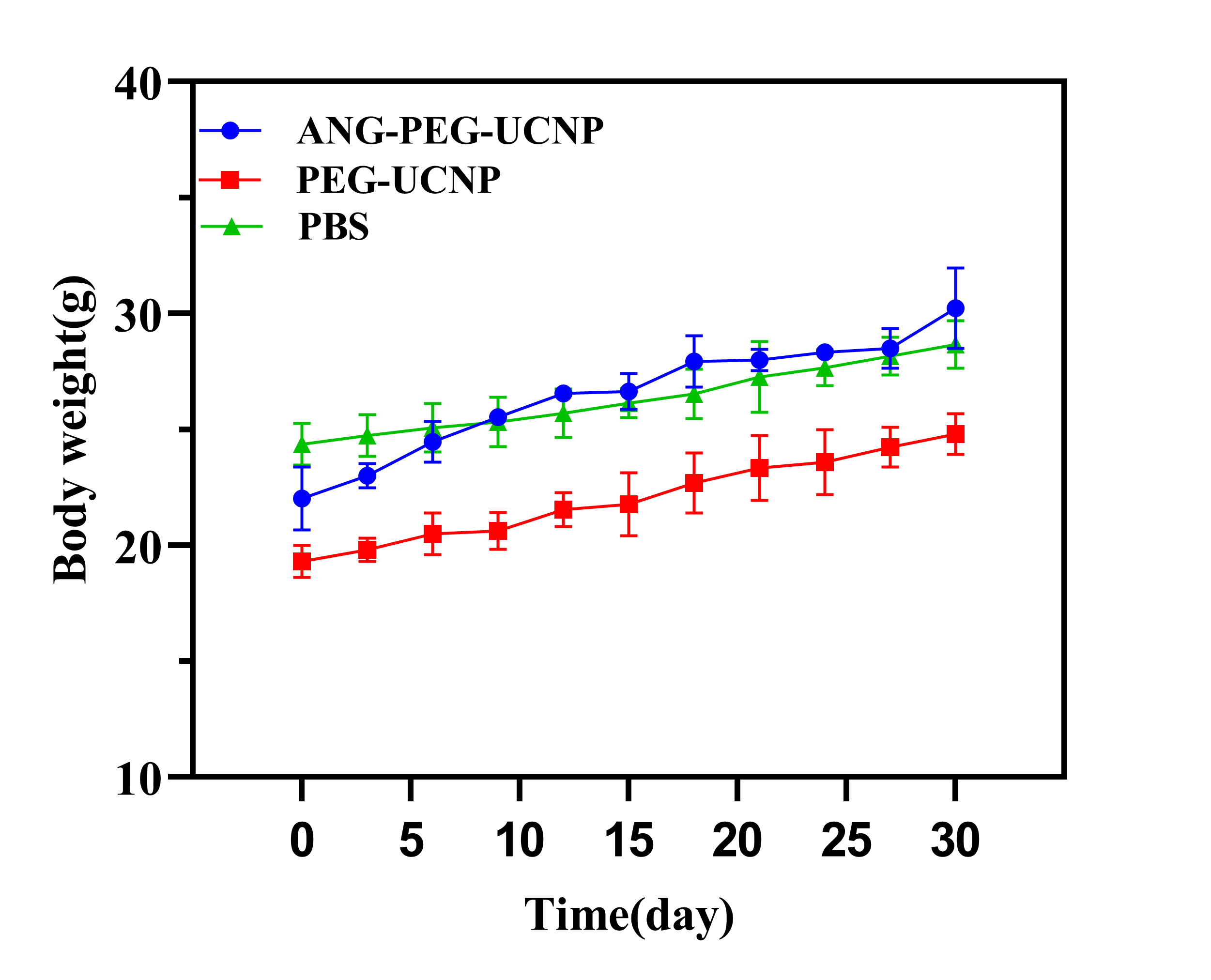
**Fig. S3** Body-weight changing of the ICR mice after intravenous injection of

ANG-PEG-UCNP, PEG-UCNP (n=3, dose=0.05 mM Gd/Kg) or PBS (n=3) at different time points. Data were expressed as mean ± standard deviation.

**
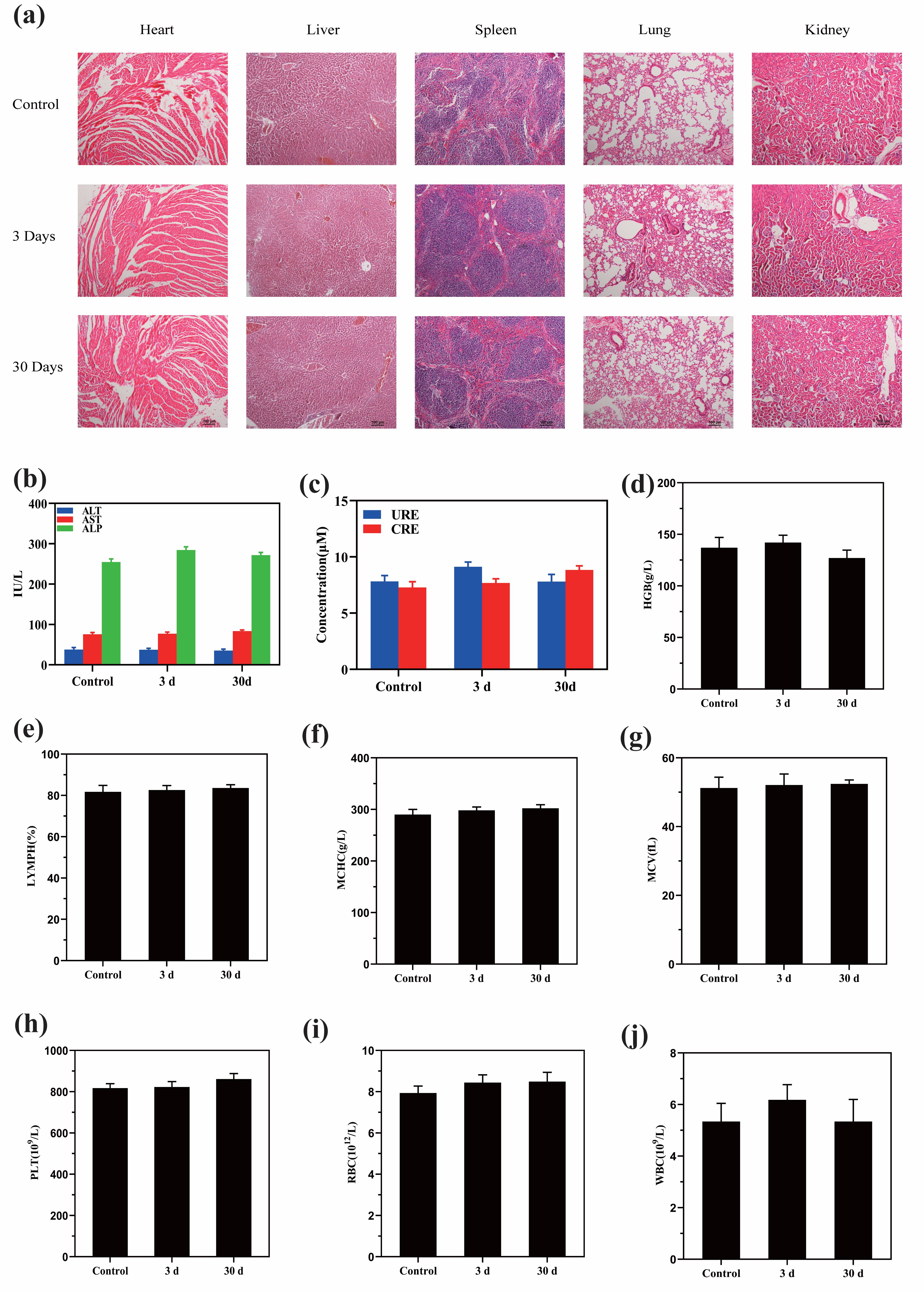
Fig. S4** Toxicity studies of ANG-PEG-UCNP in vivo. **(a)** H&E-stained tissues from mice brain to monitor the histological changes in cortex, hippocampus and striatum of brain after the intravenous injection of ANG-PEG-UCNP (15 mg Gd/kg) at difffferent time points or receiving no injection as control. Scale bar: 200 μm. **(b-j)** Blood biochemistry data obtained from mice after the intravenous injection of ANG-PEG-UCNP (n = 5, dose = 15 mg Gd/kg) at various time points or receiving no injection as control.

**Fig.S5** **(a)** Fluorescence and **(b)** Bar-graph of ANG-PEG-UCNP and PEG-UCNP on the uptake of Z310 cells by incubating for 30 min, 60 min, 90 min respectively. **(c)** Fluorescence and **(d)** Bar-graph of ANG-PEG-UCNP and PEG-UCNP on the uptake of Z310 cells at different concentration of 1500, 750, 375, 200 μg/mL, respectively.


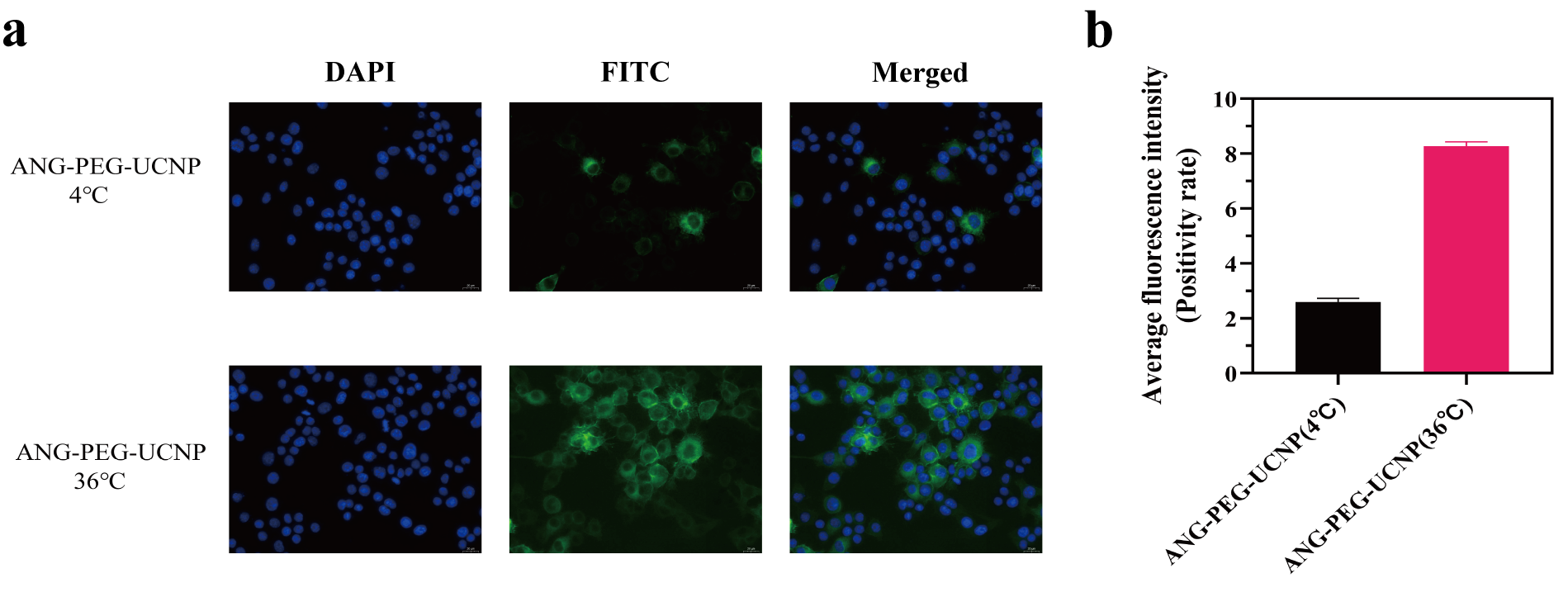


**Fig. S6** **(a)** Fluorescence of incubation temperature dependence on the uptake of ANG-PEG-UCNP and PEG-UCNP by Z310 cells at 4 ℃ and 36 ℃. **(b)** Bargraph of incubation temperature-dependent cellular uptake of ANG-PEG-UCNP and PEG-UCNP.


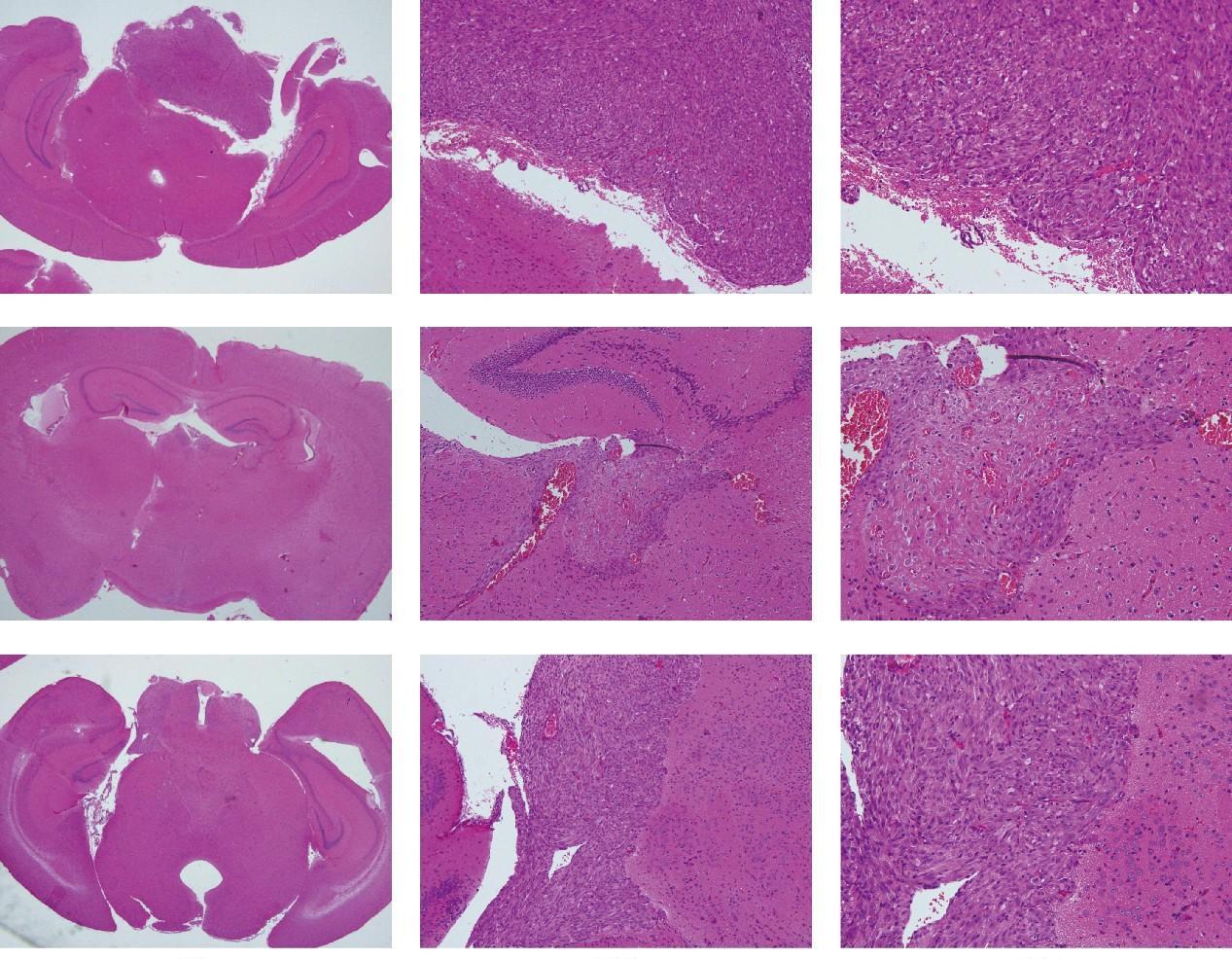


**Fig. S7** H&E-stained brain tissues from mice to confirm the successful establishment of hydrocephalus model caused by glioma. The arrows showing glioma.


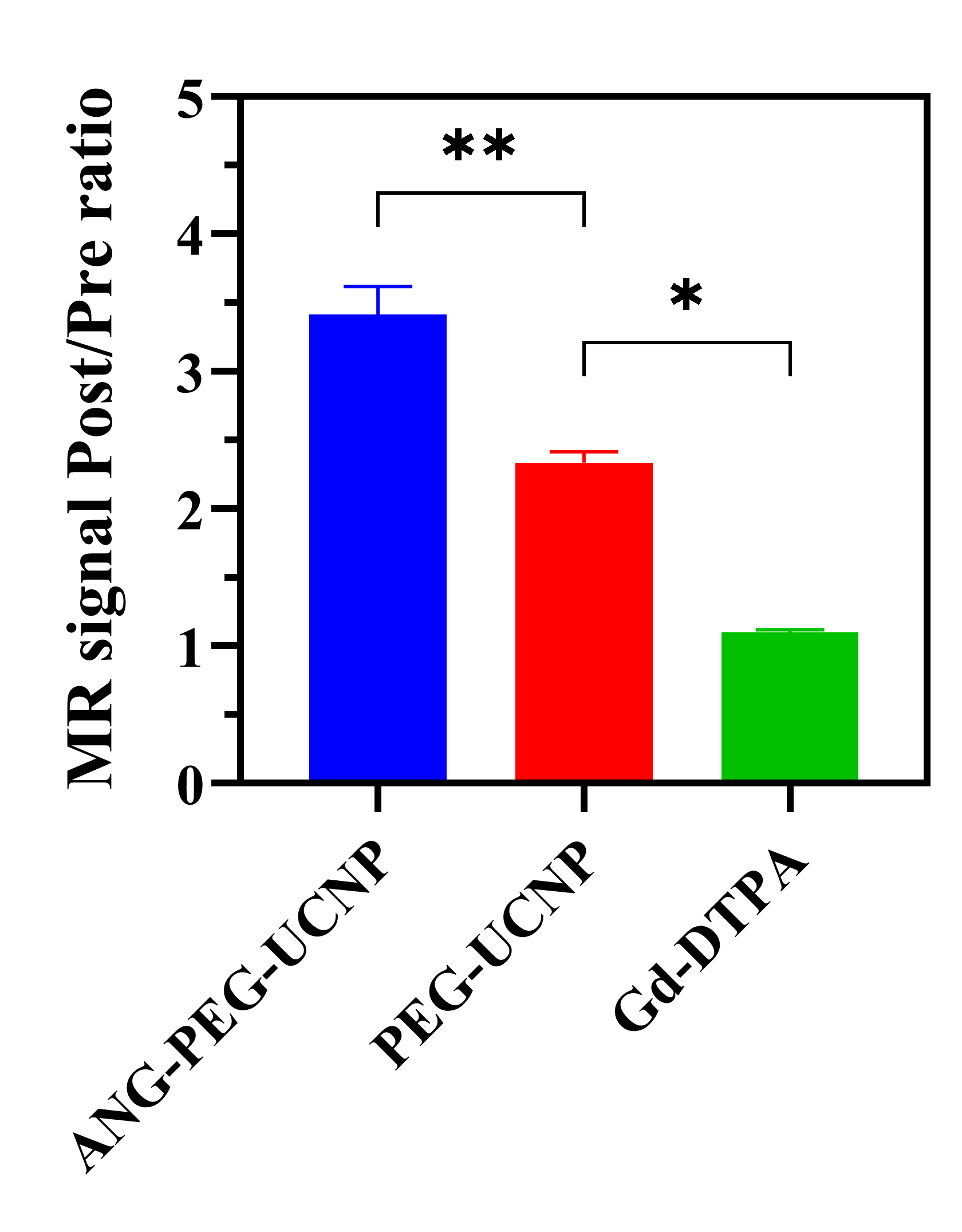


**Fig. S8** Histograms of MR signal post/pre ratios at 2 h of mice after different injections of ANG-PEG-UCNP, PEG-UCNP and Gd-DTPA, respectively. (**p*<0.05, ***p*<0.01)
